# Comparison of three culture media in assessing the sensitivity of antibiotics to common foodborne microorganisms

**DOI:** 10.25122/jml-2021-0404

**Published:** 2022-05

**Authors:** Sudad Jasim Mohammed, Adil Turki Al-Musawi, Aliaa Saadoon Al-Fraji, Hayder Shannoon Kareem

**Affiliations:** 1. Market Research and Consumer Protection Center, University of Baghdad, Baghdad, Iraq; 2. Central Public Health Laboratory, Ministry of Health, Baghdad, Iraq

**Keywords:** Antibiotic, culture media, sensitivity test, food poisoning, CLSI – Clinical Laboratory Standards Institute, MRSA– Methicillin-resistant *Staphylococcus aureus*, PSP – Pancreatic stone protein.

## Abstract

This study aimed to determine the antibiotic susceptibility of seven antibiotics, (Amoxicillin (AX), Ampicillin (AM), Chloramphenicol (C), Ciprofloxacin (CIP), Doxycycline (DO), Gentamicin (CN) and Neomycin (N)) on some common microorganisms that cause food poisoning. Furthermore, we aimed to compare three types of culture media in assessing antibiotics susceptibility. A sensitivity test was carried out using six bacterial isolates: *Micrococcus spp*., *Staphylococcus aureus, Escherichia coli, Salmonella spp*., *Proteus mirabilis, Pseudomonas aeruginosa*. These bacterial isolates were identified at the Food Microbiology Division, Public Health Laboratory using three culture media: Mueller Hinton Agar (MHA), Antibiotic Assay Medium A (AAM), and nutrient agar (NA). The results showed that all of these media are suitable to test antibiotic sensitivity. Bacterial sensitivity and resistance between these media (P≤0.01) were recorded, with significant differences found at the tested probability level.

## INTRODUCTION

Food poisoning, also known as foodborne illness, is caused by eating contaminated food. Bacterial and parasite infections and their toxins are the most common types of food poisoning, resulting from contaminated food, poor food hygiene, or neglect of health safety practices by restaurant workers [1–3]. *Escherichia coli* O157:H7 is the most dangerous contamination of red meat and its products. There is a high rate of infections in rural areas as a result of direct contact between the carcass and its skin or feces contaminated with bacteria during the slaughtering and skinning process, and the transmission of infection from one carcass contaminated with this bacteria from meat to other carcasses, as well as contamination of cutting, chopping and manufacturing devices [4]. An antibiotic sensitivity test measures how sensitive bacteria are to different antibiotics. Therefore, the antibiotic sensitivity test offers important and useful information regarding the antibiotics that must be effective against microbes [5]. Susceptibility testing allows researchers to adjust the antibiotic approach, moving from experimental treatment to direct diagnosis based on the organism's experience, knowledge, and affection, and at last, to antibiotic selection [6]. Sensitivity testing is performed in a microbiology laboratory, where culture for detecting bacterial growth is applied, and the zone of inhibition is determined [7–8]. This study aimed to assess three types of culture media in detecting bacterial sensitivity.

## MATERIAL AND METHODS

The liquid medium nutrient broth (NB) was prepared according to the manufacturer's instructions to activate and grow six bacterial isolates (*Micrococcus spp., Staphylococcus aureus, Proteus mirabilis, Pseudomonas aeruginosa, Escherichia coli, Salmonella spp*.) These microorganisms are among the common causes of diseases to which humans and animals are exposed. Consequently, test bacteria were taken from food sources obtained from the Food Microbiology Division, Central Public Health Laboratory. These microorganisms were contaminants that cause spoilage of some foods, including raw milk, pasteurized milk cheeses, and meat products diagnosed at the Food Microbiology Division, Central Public Health Laboratory. Four pure colonies with the same morphological characteristics of these test bacteria were grown on Mueller Hinton Agar (MHA), Antibiotic Assay Medium A (AAM), and nutrient agar (NA) medium supplied by Himedia (India) and transferred separately, and then incubated at a temperature of 35–37°C in test tubes containing 4–5 ml of NB medium for 18 hours. The turbidity of the bacterial growth was compared to that of a standard turbidity constant solution (McFarland's solution) by reading the optical density using a spectrophotometer at a wavelength of 450 nm after dipping a sterile swab into the standardized bacteria mix. Streaking methods were used to obtain single isolates (pure culture). The inoculation plates dried out within 5 minutes. The antibiotic discs were then placed using sterile forceps on an agar surface. Plates were incubated at 37°C for 18–24 hr. The inhibition zone was determined using a ruler for each disc [9, 10]. The antibiotic concentration and the classification in the diameter of the inhibition zones according to Clinical Laboratory Standards Institute (CLSI) are shown in Table 1 [11].

**Table 1:** Antibiotic concentration and inhibition zone diameter (Mm) testing standards for bacteria according to CLSI [11].

Antibiotics	Concentration μg	Resistant	Intermediate	Susceptible
**Amoxicillin (AX)**	30 μg	≤18	14–17	≥13
**Gentamicin (CN)**	10 μg	≤12	13–14	≥15
**Neomycin (N)**	30 μg	≤17	13–16	≥12
**Chloramphenicol**	30 μg	≤18	13–17	≥12
**Doxycycline (DO)**	30 μg	≤14	11–13	≥10
**Ampicillin (AM)**	10 μg	≤13	14–16	≥17

### Statistical analysis

Data was analyzed using SAS 2011 (Statistical Analysis System). The Chi-Square test was used to compare the percentages (probability 0.05 and 0.01) [12].

## RESULTS

The grades of the compassion test of bacterial isolates for each antibiotic were shown on each of the following media: MHA, AAM, and NA. Cultures were represented by the following microorganisms:

### 1. *Micrococcus spp*.

All isolates were hypersensitive to Neomycin (100%), Gentamicin (100%), Amoxicillin (100%) and Ampicillin (100%). Most isolates showed moderate susceptibility to Chloramphenicol (50%), Doxycycline (100%), and Ciprofloxacin (100%). This result is similar in all media (Tables 2, 3 and 4).

**Table 2:** Antibiotic sensitivity test for some bacterial isolate using Mueller Hinton Agar.

Antibiotics	Bacteria species											
	*Micrococcus* (4)		*S. aureus* (4)		*Salmonella* (4)		*E. coli* (4)		*P. aeruginosa* (4)		*Proteus mirabilis* (4)	
	S. %	R. %	S. %	R. %	S. %	R. %	S. %	R. %	S. %	R. %	S. %	R. %
**Amoxicillin (AX) 25 μg**	100	0	75	25	75	25	75	25	50	50	0	100
**Gentamicin (CN) 10 μg**	100	0	100	0	75	25	100	0	25	75	25	75
**Neomycin (N) 30 μg**	100	0	50	50	50	50	75	25	50	50	100	0
**Chloramphenicol (C) 30 μg**	50	50	75	25	100	0	50	50	75	25	50	50
**Doxycycline (DO) 30 μg**	100	0	75	25	100	0	100	0	50	50	25	75
**Ampicillin (AM) 10 μg**	100	0	25	75	75	25	0	100	25	75	0	100
**Ciprofloxacin (CIP) 5 μg**	100	0	100	0	100	0	100	0	75	25	100	0
**Chi-Square (χ^2^)**	10.50**		15.47**		16.02**		15.85**		11.73**		16.14**	

** – P≤0.01; 4 – Four well isolated colonies of the same morphological type.

**Table 3:** Antibiotic sensitivity test for some bacterial isolate using Antibiotic Assay Medium A.

AAntibiotics	Bacteria species											
	*Micrococcus* (4)		*S. aureus* (4)		*Salmonella* (4)		*E. coli* (4)		*P. aeruginosa* (4)		*Proteus mirabilis* (4)	
	S. %	R. %	S. %	R. %	S. %	R. %	S. %	R. %	S. %	R. %	S. %	R. %
**Amoxicillin (AX)**	0	100	75	25	25	75	75	25	50	50	0	100
**Gentamicin (CN)**	0	100	100	0	25	75	100	0	75	25	25	75
**Ampicillin (AM)**	0	100	25	75	25	75	0	100	75	25	0	100
**Neomycin (N)**	0	100	50	50	50	50	75	25	50	50	100	0
**Chloramphenicol (C)**	50	50	75	25	0	100	50	50	25	75	50	50
**Doxycycline (DO)**	0	100	75	25	0	100	100	0	50	50	25	75
**Ciprofloxacin (CIP)**	0	100	0	0	0	100	100	0	25	75	100	0
**Chi-Square (χ^2^)**	10.50**		15.47**		16.02**		15.85**		11.73**		16.14**	

** – P≤0.01; 4 – Four well isolated colonies of the same morphological type.

**Table 4:** Antibiotics sensitivity test for some bacterial isolate using nutrient agar.

AAntibiotics	Bacteria species											
	*Micrococcus* (4)		*S. aureus* (4)		*Salmonella* (4)		*E. coli* (4)		*P. aeruginosa* (4)		*Proteus mirabilis* (4)	
	S. %	R. %	S. %	R. %	S. %	R. %	S. %	R. %	S. %	R. %	S. %	R. %
**Amoxicillin (AX)**	0	100	75	25	25	75	75	25	50	50	0	100
**Gentamicin (CN)**	0	100	100	0	25	75	100	0	75	25	25	75
**Neomycin (N)**	0	100	50	50	50	50	75	25	50	50	100	0
**Chloramphenicol (C)**	50	50	75	25	0	100	50	50	25	75	50	50
**Ampicillin (AM)**	0	100	25	75	25	75	0	100	75	25	0	100
**Doxycycline (DO)**	0	100	75	25	0	100	100	0	50	50	25	75
**Ciprofloxacin (CIP)**	0	100	100	0	0	100	100	0	25	75	100	0
**Chi-Square (χ^2^)**	10.50**		15.47**		16.02**		15.85**		11.73**		16.14**	

** – P≤0.01; 4 – Four well isolated colonies of the same morphological type.

### 2. *Staphylococcus aureus*

Most *S. aureus* isolates were sensitive to Amoxicillin (75%), Chloramphenicol (75%), Doxycycline (75%), and Gentamicin (100%). In addition, all *S. aureus* isolates were sensitive to Ciprofloxacin (100%) and showed moderate susceptibility to Neomycin (50%), but all isolates showed resistance to Ampicillin (Tables 2, 3 and 4).

### 3. *Salmonella spp*.

Most isolates were sensitive to Ampicillin (75%), Amoxicillin (75%), and Gentamicin (75%). All isolates were susceptible to Ciprofloxacin (100%), Chloramphenicol (100%), and Doxycycline (100%). Most isolates showed moderate susceptibility to Neomycin (50%) (Tables 2, 3 and 4).

### 4. *Escherichia coli*

All isolates remained sensitive to Ciprofloxacin (100%), Doxycycline (100%) and Gentamicin (100%). Most isolates were sensitive to Amoxicillin (75%), and Neomycin (75%) and showed moderate susceptibility to Chloramphenicol (50%). However, most isolates showed resistance to Ampicillin (100%) (Tables 2, 3 and 4).

### 5. *Pseudomonas aeruginosa*

All isolates were sensitive to Ciprofloxacin (75%), Chloramphenicol (75%). Most isolates showed moderate susceptibility to Amoxicillin (50%), Neomycin (50%), and Doxycycline (50%). Most isolates showed resistance to Ampicillin (25%) and Gentamicin (25%) (Tables 2, 3 and 4).

### 6. *Proteus mirabilis*

All isolates were sensitive to Ciprofloxacin (100%) and Neomycin (100%), and most isolates showed moderate susceptibility to Chloramphenicol (50%). However, most isolates showed resistance to Doxycycline (25%), Gentamicin (25%). Additionally, all isolates showed resistance to Ampicillin (100%) and Amoxicillin (100%) (Tables 2, 3 and 4).

## DISCUSSIONS

Our results showed non-significant differences in bacterial sensitivity and resistance against different antibiotics in MHA, AAM, and NA. All these media can be used for antibiotic sensitivity tests and give satisfactory results. These results agree with those obtained by Coban [13], who recorded that AAM can be successfully used for antibiotic susceptibility tests in addition to MHA. The reason is that the emergence of bacterial strains in most cases develops multiple resistances to different types of antibiotics due to their misuse in treating various diseases.

These results were in agreement with Donkor et al. [14] who found that most *S. aureus* isolates were sensitive to Gentamicin and Chloramphenicol, while *E. coli* isolates were sensitive to Tetracycline. Moreover, most *S. aureus* isolates were sensitive to Ampicillin and Chloramphenicol because the excessive use of antibiotics has led to antibiotic resistance, and bacteria are known to be multi-resistant due to successive mutations [15].

Our result was in agreement with Bekele et al. [16] who found that Gentamicin was the most efficient antibiotic, while Amoxicillin and Tetracycline were less efficient. Our results were in agreement with the findings of others [16]. For example, Sabike et al. [17] found that the bacterial isolates used in their study were less sensitive to Ampicillin and Amoxicillin, while bacteria were sensitive to Gentamicin. A study by Vuotto et al. [18] recorded that Gentamicin was the best active medication of the 12 antibiotics tested *in vitro*.

Our results agreed with Farrell et al. [19], who mentioned that the antibiogram analysis of Gentamicin, Ciprofloxacin, Chloramphenicol, Amoxicillin, and Oxytetracycline was each 90% responsive to isolates. Most of the bacterial isolates resistant to Tetracycline were biofilm-producing due to their strong virulence factors in addition to their possession of efflux pumps [20]. Balemi et al., [21] found that *Salmonella spp*. and *S. aureus* were susceptible to Aminoglycosides and Oxytetracycline, respectively. Farrell et al. [19] found that *S. aureus* and Salmonella were Penicillin resistant (61.4% and 38.5%, respectively). Regarding the *E. coli* resistance of isolates, Ampicillin and Tetracycline were observed in 24.3% of *E. coli* isolates, 15.6%, and 13.5%. These findings agree with our findings. One of the reasons for the resistance of *E. coli* bacteria to antibiotics belonging to the quinolone group is a change in the target site and reduction in the permeability of external film of bacteria and its possession of efflux systems that include AcrAB-ToIC, MdfA, YhiV [22].

Our study agrees with Niederstebruch et al. [23], who found that bacterial isolates showed resistance to antibacterial agents, being sensitive to Ciprofloxacin and Gentamicin.

Furthermore, another study found that *E. coli* and *S. aureus* were sensitive to Gentamicin, while *S. aureus* and *E. coli* resistant to Penicillin [21].

The pathogenic bacteria *P. aeruginosa, E. Coli*, and *Proteus mirabilis* are gram-negative bacteria, while *S. aureus, Micrococcus spp*., and *Salmonella spp*. are gram positive, which resist the methicillin group (MRSA). The secondary metabolites of pancreatic stone protein (PSP) can inhibit the activities of gram negative bacteria that have lipopolysaccharides (LPS) and play a role in inhibiting beta lactamase enzymes produced by *S. aureus* [24, 25]. Our results was in accordance with those reported in previous studies in which the most commonly reported species were *E. coli, P. aeruginosa*. [26].

## CONCLUSIONS

This study compared the sensitivity and antibiotic resistance using three culture media against several bacterial isolates that cause common foodborne illness. Future work should focus on the suitable media for bacterial isolates.
